# Supplementary material for: A logic-based dynamic modeling approach to explicate the evolution of the central dogma of molecular biology
Source: PLoS One. 2017 Dec 21;12(12):e0189922. doi: 10.1371/journal.pone.0189922 (PMC5739447; doi:10.1371/journal.pone.0189922)
Supplement: S1 File — (DOCX) [file pone.0189922.s001.docx]

**Model reconstruction**

Central dogma is a generalized model of gene expression regulation. Therefore, a bottom-up approach was used to reconstruct the new central dogma model. Therefore, we have abstracted several metabolic and signaling pathways in KEGG databases and verified the primary model using the recent reviews about the gene expression regulation [1-4]. Then, a team of biological and mathematical researchers have reviewed and criticized the model. We have tried to reconstruct as general and possible model of current understanding of the central dogma in different organisms of plants, nematodes and mammals.

Our model has 7 nodes instead of former 4-node model. **Activator**, **DNA**, **mRNA** and **Protein** are shared nodes in both models but **DegProtein**, **miRNA** and **DegRNA** are newly added nodes. Activator is a chemical molecule or a residue which facilitate the activation of a protein. DegRNA is considered as a protein complex which degrades RNA molecules. DNA is a symbol of active (non-silenced) genes which are transcribed by RNA polymerase. miRNAs are RNA species involved in regulation of gene expression (silencing of target genes). In order to simplify the model, all other RNA species which has negative effect on protein production is shown with the same label as miRNAs. mRNA is a mature messenger RNA that is ready for translation. Again for simplification, all other RNA species which has positive effect on protein production is shown with the same label as mRNA. Protein is a functionally active protein. Finally, Ubiquitin is a symbol of Ubiquitination as a post-translational modification involved in negative regulator of protein amount.

Logical roles are more intensely modified in 7-node model in comparison to 4-node model. In the 4-node model, V1 and V2 are “Activatory gene expression” in which DNA code is transcribed and translated into a functional protein. Then, V2 is “Inhibitory gene expression” in which transcription is inhibited and gene expression is downregulated.

V1: Activatory gene expression

V2: Inhibitory gene expression

This simple representation of the Central Dogma has now more resolution in each of two mentioned versions. Our current understanding of interaction in cellular systems in more detailed including:

V1: Activatory

V2: mRNA expression inhibition

V3: miRNA expression Inhibition

V4: Gene silencing

V6: Gene silencing & miRNA expression inhibition

V5: Gene silencing & mRNA expression inhibition

V7: miRNA & mRNA expression inhibition

V8: Inhibitory

All the Boolean functions are derived manually from references mentioned below. Some of the functions were info-graphically abstracted in conceptual Figures published in these references. For example, Francis Crick [5] showed the information flow between DNA, RNA and Protein with arrows (see Figures 1-3 in the paper) and some simple formulations for the first time. Other functions have been mentioned within the text of publications which were extracted to reconstruct the logical functions. For instance, the selected text below was used to extract a part of extracted miRNA related functions [3]:

“*It was first believed that small RNAs especially miRNAs can only induce transcriptional and post-transcriptional silencing levels. During PTGS, unceasing gene expression occurs and just target mRNA is degraded, but in the case of epigenetic silencing (also called transcriptional gene silencing (TGS)) expression is thoroughly inhibited in a potent and long lasting method. Technically, both ways are available for scientists, but what about inducing or increasing transcription rate of target genes by a very similar method? Fascinatingly, it is identified that small RNAs can also induce expression of target genes. Many mature miRNAs translocate back to the nucleus for TGS or activation-targeted promoter sequences (Liao et al. 2010; Younger et al. 2009; Kim et al. 2008)*.”

In the following, another version of Tables 1 and 2 in the manuscript is presented along with relevant references used for each function. Note that we aimed to formulate the Central Dogma as a law of information transfer in biological systems similar to Crick’s approach [5]. Hence, these logical functions are not equivalent to any biochemical reaction pathway.

**Table 1:**

|  |  | *Logical rule* | | References |
| --- | --- | --- | --- | --- |
| **Node** | ***Definition*** | V1: Activatory gene expression | V2: Inhibitory gene expression |  |
| Activator | The chemical molecules which facilitate active protein generation before or after translation | Activator | | [5, 6] |
| DNA | The genes transcribed to coding RNA | DNA | | [5, 6] |
| mRNA | The coding RNA | DNA & Protein | DNA & !Protein | [5, 6] |
| Protein | The functional active protein | (mRNA & Activator) \| Protein | | [5, 6] |

**Table 2:**

|  |  | *Logical rule* | | | | | | | | References |
| --- | --- | --- | --- | --- | --- | --- | --- | --- | --- | --- |
| **Node** | ***Definition*** | V1: Activatory | V2: mRNA expression inhibition | V3: miRNA expression Inhibition | V4: Gene silencing | V5: Gene silencing & mRNA expression inhibition | V6: Gene silencing & miRNA expression inhibition | V7: miRNA & mRNA expression inhibition | V8: Inhibitory |  |
| Activator | The chemical molecules or residue which facilitate active protein generation before or after translation | Protein \| Activator | | | | | | | | [5, 6] |
| DegProtein | All biochemical modifications that are involved in negative regulation of protein amount | Protein & !DegProtein | | | | | | | | [7-9] |
| DegRNA | The protein complex which degrades RNA species | miRNA & Protein & !DegProtein | | | | | | | | [1-4, 10] |
| DNA | The non-silenced genes which is detectable by RNA polymerase | (miRNA & Protein) \| DNA | | | (**!miRNA** & Protein) \| DNA | | | (miRNA & Protein) \| DNA | (**!miRNA** & Protein) \| DNA | [1-6] |
| miRNA | A RNA species which is involved in regulation of gene silencing (for simplification, all other RNA species which has negative effect on protein production is shown with the same label) | (DNA & Protein \| mRNA) & !DegRNA | | (DNA & **!Protein** \| mRNA) & !DegRNA | (DNA & Protein \| mRNA) & !DegRNA | (DNA & **!Protein** \| mRNA) & !DegRNA | (DNA & Protein \| mRNA) & !DegRNA | (DNA & **!Protein** \| mRNA) & !DegRNA | | [1-4, 7, 8] |
| mRNA | The mature messenger RNA that is ready for translation (for simplification, all other RNA species which has positive effect on protein production is shown with the same label) | DNA & Protein & !DegRNA | DNA & **!Protein** & !DegRNA | DNA & Protein & !DegRNA | DNA & Protein & !DegRNA | | DNA & **!Protein** & !DegRNA | | | [1-3, 5-8] |
| Protein | The functional active protein | (mRNA & Activator \| Protein) & !DegProtein | | | | | | | | [5-9] |

Additionally, to assign probability values to Boolean functions in the probabilistic version of the model, we obtained estimates of these values according to a text-mining approach by identifying the frequency of related keywords in three independent scientific search engines, namely PubMed, GoogleScholar and Scopus. For instance, as shown in Table 3, the estimated probability values of miRNA activation were calculated based on the ratio of opposite keywords (i.e. P=activation/(inhibition & suppression)). The mean P of 0.11 (~0.1) was then used in the probabilistic version of the model.

**Table 3:**

|  | **PubMed** | **Google Scholar** | **Scopus** |
| --- | --- | --- | --- |
| **miR* AND inhibits** | 4047 | 442000 | 5272 |
| **"miR* inhibits"** | - | 23800 | 81 |
| **miR* AND suppresses** | 2265 | 165000 | 2853 |
| **"miR* suppresses"** | - | 16100 | 41 |
| **miR* AND activates** | 507 | 81400 | 977 |
| **"miR* activates"** | - | 1520 | 7 |
| **Ratio** | 0.08 | 0.13 | 0.12 |

Note that the quotation symbol is not meaningful for the PubMed search engine.

The counts were retrieved on 8^th^ Nov 2017.

**References:**

1. Jamalkandi SA, Masoudi-Nejad A. RNAi pathway integration in Caenorhabditis elegans development. Functional & integrative genomics. 2011;11(3):389.

2. Jamalkandi SA, Masoudi-Nejad A. Reconstruction of Arabidopsis thaliana fully integrated small RNA pathway. Functional & integrative genomics. 2009;9(4):419.

3. Jamalkandi SA, Azadian E, Masoudi-Nejad A. Human RNAi pathway: crosstalk with organelles and cells. Functional & integrative genomics. 2014;14(1):31-46.

4. Borna H, Imani S, Iman M, Azimzadeh Jamalkandi S. Therapeutic face of RNAi: in vivo challenges. Expert opinion on biological therapy. 2015;15(2):269-85.

5. Crick F. Central dogma of molecular biology. Nature. 1970;227(5258):561-3. doi: 10.1038/227561a0.

6. Jacob F, Monod J. Genetic regulatory mechanisms in the synthesis of proteins. Journal of molecular biology. 1961;3:318-56.

7. Milo R, Phillips R. Cell biology by the numbers. New York, NY: Garland Science, Taylor & Francis Group; 2016. xlii, 356 pages p.

8. Systems Biology for Signaling Networks. New York, NY: Springer New York; 2010.

9. Koonin EV. Does the central dogma still stand? Biology direct. 2012;7(1):27-. doi: 10.1186/1745-6150-7-27.

10. Catalanotto C, Cogoni C, Zardo G. MicroRNA in Control of Gene Expression: An Overview of Nuclear Functions. Int J Mol Sci. 2016;17(10). doi: 10.3390/ijms17101712. PubMed PMID: 27754357; PubMed Central PMCID: PMCPMC5085744.
